# Relationships between peak alpha frequency, age, and autistic traits in young children with and without autism spectrum disorder

**DOI:** 10.3389/fpsyt.2024.1419815

**Published:** 2024-08-30

**Authors:** Masafumi Kameya, Tetsu Hirosawa, Daiki Soma, Yuko Yoshimura, Kyung-min An, Sumie Iwasaki, Sanae Tanaka, Ken Yaoi, Masuhiko Sano, Yoshiaki Miyagishi, Mitsuru Kikuchi

**Affiliations:** ^1^ Department of Psychiatry and Neurobiology, Graduate School of Medical Science, Kanazawa University, Kanazawa, Japan; ^2^ Research Center for Child Mental Development, Kanazawa University, Kanazawa, Japan; ^3^ Faculty of Education, Institute of Human and Social Sciences, Kanazawa University, Kanazawa, Japan; ^4^ Centre for Human Brain Health, School of Psychology, University of Birmingham, Birmingham, United Kingdom; ^5^ Department of Psychology, Faculty of Liberal Arts, Teikyo University, Tokyo, Japan

**Keywords:** autism spectrum disorder, peak alpha frequency, magnetoencephalography, neurodevelopment, brain regions

## Abstract

**Background:**

Atypical peak alpha frequency (PAF) has been reported in children with autism spectrum disorder (ASD); however, the relationships between PAF, age, and autistic traits remain unclear. This study was conducted to investigate and compare the resting-state PAF of young children with ASD and their typically developing (TD) peers using magnetoencephalography (MEG).

**Methods:**

Nineteen children with ASD and 24 TD children, aged 5-7 years, underwent MEG under resting-state conditions. The PAFs in ten brain regions were calculated, and the associations between these findings, age, and autistic traits, measured using the Social Responsiveness Scale (SRS), were examined.

**Results:**

There were no significant differences in PAF between the children with ASD and the TD children. However, a unique positive association between age and PAF in the cingulate region was observed in the ASD group, suggesting the potential importance of the cingulate regions as a neurophysiological mechanism underlying distinct developmental trajectory of ASD. Furthermore, a higher PAF in the right temporal region was associated with higher SRS scores in TD children, highlighting the potential role of alpha oscillations in social information processing.

**Conclusions:**

This study emphasizes the importance of regional specificity and developmental factors when investigating neurophysiological markers of ASD. The distinct age-related PAF patterns in the cingulate regions of children with ASD and the association between right temporal PAF and autistic traits in TD children provide novel insights into the neurobiological underpinnings of ASD. These findings pave the way for future research on the functional implications of these neurophysiological patterns and their potential as biomarkers of ASD across the lifespan.

## 1 Introduction

Autism spectrum disorder (ASD), as defined by the American Psychiatric Association (1), is a group of neurodevelopmental disorders characterized by challenges in social communication and interaction, alongside restricted and repetitive behaviors. These symptoms, which typically emerge in infancy, persist throughout life. With the increasing prevalence of ASD and its significant socioeconomic impact (2), identifying its unique and reliable biomarkers has become crucial to public health. As highlighted by Eapen et al. (3), these biomarkers are particularly vital for developing personalized therapies.

Previous traditional imaging studies on ASD were predominantly conducted using task-based methods. However, given the clinical heterogeneity of ASD, reliance on task performance may pose limitations that affect the universality and applicability of identified biomarkers. In addition, task-based assessments can be time-consuming, adding to the burden on patients and clinicians. In contrast, analysis of resting-state data is a promising alternative to task-based assessments. This is because resting-state data analysis bypasses the abovementioned limitations and potentially identifies more consistent biomarkers applicable across diverse ASD populations, thereby paving the way for the development of more effective and personalized interventions.

Neuroimaging technologies have offered new insights into the biological mechanisms underlying ASD. Among the various existing imaging modalities, electroencephalography (EEG) stands out for its application in pediatric research. Unlike positron emission tomography or computerized tomography, which involve radiation, and magnetic resonance imaging (MRI), which can be loud, EEG is non-invasive and silent, making it particularly suitable for studying children, both with and without ASD. EEG studies have revealed patterns potentially linked to ASD’s hallmark difficulties in social communication. A key focus has been on alpha oscillations (8–12 Hz), the predominant rhythms observed during wakefulness, especially in the eyes-closed condition, as alpha power diminishes when the eyes are open and visual input is received (4). Alpha oscillations have been associated with social coordination (5, 6) and information processing across thalamocortical and cortico-cortical networks (7–10). However, research on alpha power in ASD presents a conundrum; findings vary widely, ranging from reports of decreased (11–13) or increased (14) alpha power compared to typically developing (TD) individuals, to reports of no significant differences between individuals with ASD and their TD peers (15, 16). These inconsistencies could be attributed to various reasons, including variations in study populations and methodological approaches (17).

Peak alpha frequency (PAF) is an important measure in the field of resting-state (RS) alpha oscillations. PAF indicates the frequency at which RS alpha oscillations attain maximum power. PAF is a clinically significant measure, as evidenced by its high heritability (18, 19); twin studies estimate its heritability at 0.81, suggesting minimal environmental influence (20). In TD individuals, a higher PAF is correlated with improved cognitive performance, including enhanced working memory and faster information processing (21–23), and is linked to higher IQ test scores (24). Additionally, PAF is associated with neurodevelopmental delays (25, 26). This significance can be attributed to the inherent temporal structuring of the brain through neural activity, where alpha rhythms are pivotal for intra- and inter-regional brain communication (4). Furthermore, PAF undergoes notable changes with age, which indicate that it is a reliable indicator of brain maturation in TD individuals (27, 28). Studies have documented a gradual shift from an 8-9 Hz alpha peak in young children (5–7 years old) to an ‘adult-like’ 10–12 Hz PAF that manifests at approximately 15 years of age (29, 30), reflecting the increasing complexity of cortical organization with age. Consequently, PAF is recognized as a sensitive measure of alpha oscillatory development (31) and is reported to provide a more precise assessment of alpha oscillatory activity than alpha power alone (32). Considering the fundamental role of alpha rhythms in various brain processes, investigating alpha rhythms in neurodevelopmental disorders such as ASD is particularly pertinent.

Several EEG and magnetoencephalography (MEG) studies have demonstrated differences in PAF between younger individuals with ASD and their TD peers (14, 16, 25, 26, 33–37). For instance, Dickinson et al. (25) found that TD children (aged 5.97 ± 2.21 years) exhibited higher PAF than children with ASD (aged 5.78 ± 2.01 years), particularly in the frontal and central regions, using EEG with an eyes-open condition involving the display of bubbles on a computer screen. Conversely, Shen et al. (33) reported that children with ASD (aged 7.8 ± 0.8 years) showed higher PAFs than TD children (aged 7.5 ± 0.8 years) using MEG under an eyes-closed condition. Edgar et al. (26) analyzed resting state eyes-closed data and showed significant PAF differences (higher PAF in children with ASD than in TD children) only in children younger than 10 years. However, no study on participants with a mean age of over 10 years has indicated a significant difference in PAF between TD and ASD populations (14, 16, 34–36). Additionally, Leno et al. (37) found no significant PAF differences at 12 months of age in children with and without a familial risk of ASD. These results suggest possible age- or experimental condition-dependent differences in PAF between children with ASD and TD children.

In addition to potential age-dependent group differences in PAF, the relationship between age and PAF may vary by diagnosis. In the TD population, PAF is positively correlated with age, beginning as early as 12 months, regardless of familial ASD risk (37), through childhood (5-10 years) (16, 25, 26, 33, 36) and into adolescence (approximately 16 years) (35). In contrast, in children with ASD, this association is reported to be non-significant during early developmental stages (6-10 years old) (25, 26, 33, 34, 36) and becomes significant only in later developmental stages, such as adolescence (35). This ASD status-specific relationship between PAF and age across developmental stages might indicate potential differences in neural maturation between TD and ASD populations. However, these results should be interpreted cautiously because the ASD group was on medication in the abovementioned studies (25, 26, 34, 35, 38). In this regard, Shen et al. (33) did not observe a significant Diagnosis-age interaction in medication-free participants approximately 7 years old, whereas Dickinson et al. (25) reported a significant Diagnosis-age interaction within a similar age range in participants where the ASD group was on medication. In addition to considering medication status, it should also be noted that experimental conditions and participant characteristics vary in the abovementioned studies, such as visual stimuli versus eyes-closed conditions, IQ profiles, and sex ratios.

The fact that individuals with ASD exhibit differences in PAF compared to their TD peers (14, 16, 25, 26, 33–37) suggests a possible connection between PAF and the severity of autistic symptoms. While the link between higher PAF and better non-verbal IQ (NVIQ) outcomes is well established—observable from as early as 24 months and persisting from childhood to adulthood in both TD and ASD populations (25, 26, 33, 34, 39)—the potential relationship between PAF and autistic symptoms remains underexplored, particularly in younger populations. To date, few studies have been conducted to directly examine the relationship between PAF and autistic symptoms. One notable study by Dickinson et al. (34) included adult participants (78 TD individuals, aged 32.5 ± 12.3 years and 93 individuals with ASD, aged 30.4 ± 13.6 years). The results indicated that in the non-ASD group, ASD symptoms (quantified by ADOS scores) were significantly negatively correlated with PAF, a relationship that remained significant after controlling for age and NVIQ. However, in the ASD group, ASD symptoms were not associated with PAF. While this lack of association in adults with ASD might suggest that PAF does not directly reflect symptom severity in this population, it raises intriguing questions, based on findings in non-ASD adults, about whether a relationship does exist between PAF and autistic traits earlier in development in children diagnosed with ASD. Since differences in PAF have been reported between TD and ASD populations in younger age groups (25, 26, 33), this study seeks to determine whether a relationship between PAF and autistic traits can be identified in young children. If such a correlation exists, it could provide evidence of a developmental link between alpha oscillations and autism that might evolve or diminish with age. Therefore, we conducted this study to explore the relationship between PAF and autistic symptoms in young children with ASD and their TD peers. To approximate a resting-state condition, we used an eyes-open setup in a dark room with a small fixation cross, ensuring minimal visual input while keeping participants’ heads stationary. This method differs slightly from the eyes-closed condition used in other studies but has shown good reliability for obtaining resting-state alpha measures in similar populations (see Methods section for details).

## 2 Methods

### 2.1 Study design and participants

In this prospective, observational study, we recruited children with ASD and TD children aged 5 to 7 years and obtained their MEG recordings in a resting-state condition. To ensure the validity and reliability of our findings, we excluded children who were taking medication and those with evident intellectual disabilities. Consequently, this study focuses on a narrow segment of the ASD population, specifically young children without intellectual disabilities or medication influences. While these criteria help maintain a controlled sample, they also limit the generalizability of our findings to the broader ASD population.

PAF was obtained using MEG, during which participants focused on a fixation cross projected onto a screen in a dark room to approximate a resting-state condition. Edgar et al. (40) reported good reliability for resting-state eyes-closed and dark room (with no visual stimulation) peak alpha frequency in TD children and those with ASD, indicating the dark room exam as a viable method to obtain resting-state alpha measures in these populations. Our experimental condition differed slightly, as we collected data with a small fixation cross in a dark room, introducing minimal visual input. We chose this procedure to ensure the children kept their heads stationary during the recordings, as staring at the fixation cross helps them stay still. Given the younger age range of our participants, it was necessary to strike a balance between the ideal condition (eyes-closed or eyes-open in a completely dark room) and practical considerations to minimize visual stimulation while ensuring the children remained still. Considering the experimental setup of this study, which is similar to that used by Shen et al. (33)―MEG in an eyes-closed condition, an average age of approximately 7.5 years, and exclusion of participants with intellectual disabilities and those taking medications―our hypotheses are twofold. First, we anticipated replicating the findings of Shen et al. (33), specifically that children with ASD would exhibit higher PAF than TD children within this age range and that higher PAF would be significantly associated with older age only within the TD population. Second, considering the higher PAF observed in the ASD population in the study by Shen et al. (33), we hypothesized a significant association between more severe autistic symptoms and higher PAF in both groups.

The clinical group comprised of 21 children diagnosed with ASD, recruited from Kanazawa University and its affiliated hospitals. ASD diagnoses were based on the DSM-IV criteria (41) and confirmed by experienced psychiatrists and psychologists using the Diagnostic Interview for Social and Communication Disorders (DISCO) (42) and/or Autism Diagnostic Observation Schedule-2 (ADOS-2) (43, 44). Some participants in the ASD group were diagnosed using only the ADOS-2, some using only the DISCO, and others using both. The control group comprised of 25 TD children with no known behavioral or language difficulties. Children who were blind and/or deaf, had other neuropsychiatric disorders, or were on an ongoing medication regimen ((including any type of medication) were excluded. Family history of ASD was not screened for in the control group. Written informed consent was obtained from the parents of the children prior to their participation in the study. The research methods and procedures were approved by the Ethics Committee of Kanazawa University Hospital, and the study was conducted in accordance with the principles outlined in the Declaration of Helsinki. This research is a part of a broader project known as the Bambi Plan at the Kanazawa University Research Center for Child Mental Development (https://kodomokokoro.w3.kanazawa-u.ac.jp/en/). It is important to note that although some participants in this study were also included in our previous research (45), there is no overlap in the results presented. Moreover, the objectives and emphases of the previous studies differ significantly from those of the present study.

### 2.2 MEG

MEG data were recorded using a 151-channel Superconducting Quantum Interference Device (SQUID) whole-head coaxial gradiometer MEG system (PQ 1151R; Yokogawa/KIT, Kanazawa, Japan) (46) within a magnetically shielded room (Daido Steel Co., Ltd., Nagoya, Japan).

We utilized a custom-made, child-sized MEG system designed to optimize the placement of sensors on children’s heads, which are generally smaller than those of adults. This design not only facilitates effective positioning but also limits head movements. Recordings were obtained at a 2,000 Hz sampling rate and low-pass filtered at 500 Hz.

We strived to keep the children stationary during recordings; however, achieving extended stillness was particularly challenging for those with ASD. Given these challenges, we determined a compromise for the minimum recording duration. We established a recording period of 130 seconds, aiming to secure a baseline of 50 seconds and allowing for some buffer time. This decision was grounded in existing research suggesting that 38 seconds of artifact-free data provides a reliable threshold for assessing spontaneous EEG characteristics such as PAF (25, 47).

During the recording, the participants laid supine in a resting state, focusing on a fixation cross mark projected onto a screen. Their eyes remained open throughout the recording. All recording sessions were scheduled between 11 am and 3 pm, and no signs of drowsiness were evident in the MEG waveforms of any child.

### 2.3 Assessment of intelligence and the severity of autism symptoms

The Social Responsiveness Scale (SRS) was used for the assessment of autism symptoms. This 65-item scale allows for the assessment of children in natural social contexts, reflecting observations over weeks or months (48). It comprises five subscales: social awareness, social cognition, social communication, social motivation, and autistic mannerisms. Higher scores indicate greater severity of autism symptoms. In our study, the SRS was completed by one of the child’s parents. Intelligence was assessed using the Kaufman Assessment Battery for Children (K-ABC). In the K-ABC, the Mental Processing Scale interprets problem-solving abilities as intelligence, whereas the Achievement Scale measures knowledge of facts (49, 50). These scores are standardized according to age, with a mean of 100 and a standard deviation (SD) of 15.

### 2.4 Magnetic resonance imaging

Structural brain images were acquired using a 1.5 T MRI scanner (SIGNA Explorer; GE Healthcare, USA). The T1-weighted gradient echo and Silenz pulse sequence were utilized (TR = 435.68 ms, TE = 0.024 ms, flip angle = 7°, FOV = 220 mm, matrix size = 256 ×256 pixels, slice thickness = 1.7 mm; a total of 130 transaxial images). This provided the necessary anatomical reference for this study. Obtaining MRI images from children aged 5-7 years can be challenging; however, due to the shorter recording time afforded by the optimized protocol, all participants successfully completed the MRI procedure.

### 2.5 Co-registration of MEG on MRI images

Co-registration of MEG and MRI images was based on specific marker locations. Four distinct markers were identified on both MEG and MRI: the midline frontal, vertex, and bilateral mastoid processes. Magnetic field-generating coils served as the markers for MEG, whereas lipid capsules acted as markers for MRI, owing to their distinct appearance as high-intensity regions. Additionally, points on the mastoid processes, nasion, and skull surface were visually pinpointed on the MRI images. Typically, 15–25 points were marked for each participant.

### 2.6 Preprocessing of MEG data

MEG data preprocessing was conducted according to the guidelines of the Organization for Human Brain Mapping (51). First, the data were downsampled to 500 Hz, and three sensors were excluded because of their poor signal quality. Second, notch filters were applied at 60, 120, and 180 Hz to remove power-supply noise, followed by the application of a band-pass filter (0.5–200 Hz). Third, independent component analysis was conducted to remove blink and cardiac artifacts. Finally, segments containing apparent motion noise or radiofrequency interference were excluded from the analyses after visual identification by an MEG expert blinded to the identities of the participants. Data were segmented into continuous segments of 5 s, with a minimum of 10 segments (50-s recording period) accepted for each participant.

### 2.7 Atlas-guided source reconstruction and segmenting

We performed signal source estimation using the original anatomies of the participants. An anatomically constrained MEG approach was used to estimate the brain signal sources (52). When estimating sources, the recorded brain activity of each participant was assumed to be located within the cortical mantle. A head model was computed using the overlapping spheres algorithm (53) with the default source space (a lower-resolution cortical surface representation with 15,000 vertices). In addition, weighted minimum-norm estimation was used to estimate source orientation constraints (54). An identity matrix was used as the noise covariance because no noise recording was available. Signal sources were grouped into 68 regions represented in Desikan-Killiany atlases (55). Principal component analysis was used to group the sources.

### 2.8 Computing spectral power

To compute the spectral power of the 68 regions defined using the Desikan-Killiany brain atlas, we used Welch’s method, which was implemented. Each epoch (5 s) was processed using a Hamming window with an 80% overlap. The resulting power spectra had an approximately 0.2 Hz frequency resolution.

### 2.9 Measurement of peak alpha frequency

**Figure 1:**
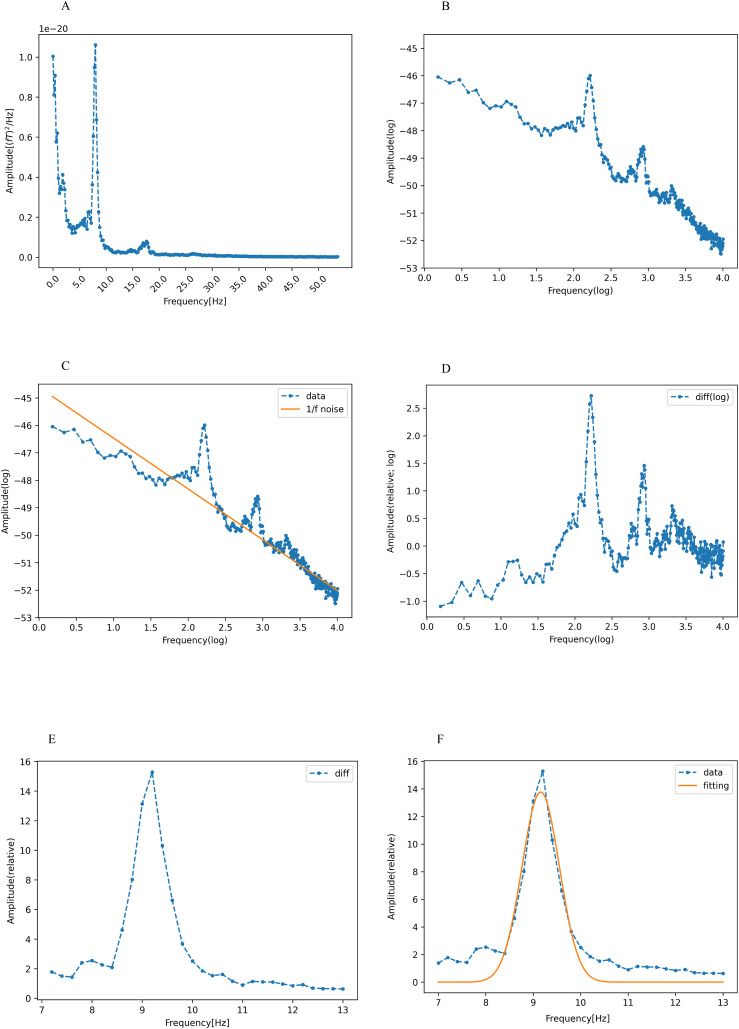
Calculation of peak alpha frequency (PAF). **(A)** The spectral density of the signal sources was estimated to be localized in the left caudal anterior cingulate cortex, showing a power spectral density in the range of 1-55 Hz. The horizontal and vertical axes represent the frequency and absolute power values, respectively. **(B)** Logarithmic transformation of frequency and absolute power values within the same range (blue dotted line). **(C)** Robust linear regression (Huber’s method, H=1.35) was applied to the log-transformed data indicated in **(B)** (dotted line). The linear regression showed a 1/f trend (orange line). **(D)** Log-transformed data (blue dotted line in **C**) minus the 1/f trend (orange line in **C**). **(E)** Both power and frequency are exponentiated in **(D)**, with the alpha band isolated to the frequency range of 7-13 Hz. **(F)** Gaussian function fitting of the residual (orange line) shown in **(E)**. The frequency of the vertex of this curve is defined as the peak alpha frequency (PAF). These figures were created using the Matplotlib library (72) in Python.

**Figure 1A** depicts the typical power spectrum density of a signal source, which was estimated to be localized to the left caudal anterior cingulate. The PAF of this signal source was derived according to established protocols from previous research (25, 35).

Our preliminary step was to counteract the inherently dominant 1/f trend often observed in EEG/MEG power spectra (56), which potentially obscures nuanced alpha peaks. This adjustment ensures that the spectral peak is accurately identified without undue emphasis on the lower alpha frequency spectrum. To neutralize the 1/f trend, we performed a log-transformation of both the frequency and power within the range of 1-55 Hz (**Figure 1B**). Consequently, the 1/f component in the original power spectrum was represented with a linear dependence on the log-frequency (**Figure 1C**).

Robust linear regression, performed using Huber’s method with M set at 1.35, was employed for the prediction of log power using log frequency. This approach is known for its resilience against outliers, particularly when juxtaposed with the traditional least squares method (57, 58). The 1/f trend, depicted by the yellow line in **Figure 1C**, was then subtracted from the original data, which is represented by the blue curve in the same figure. The resulting residual values were exponentiated, and the alpha band was isolated in the 7-13 Hz range (26) (**Figure 1D**).

**Figure 2:**
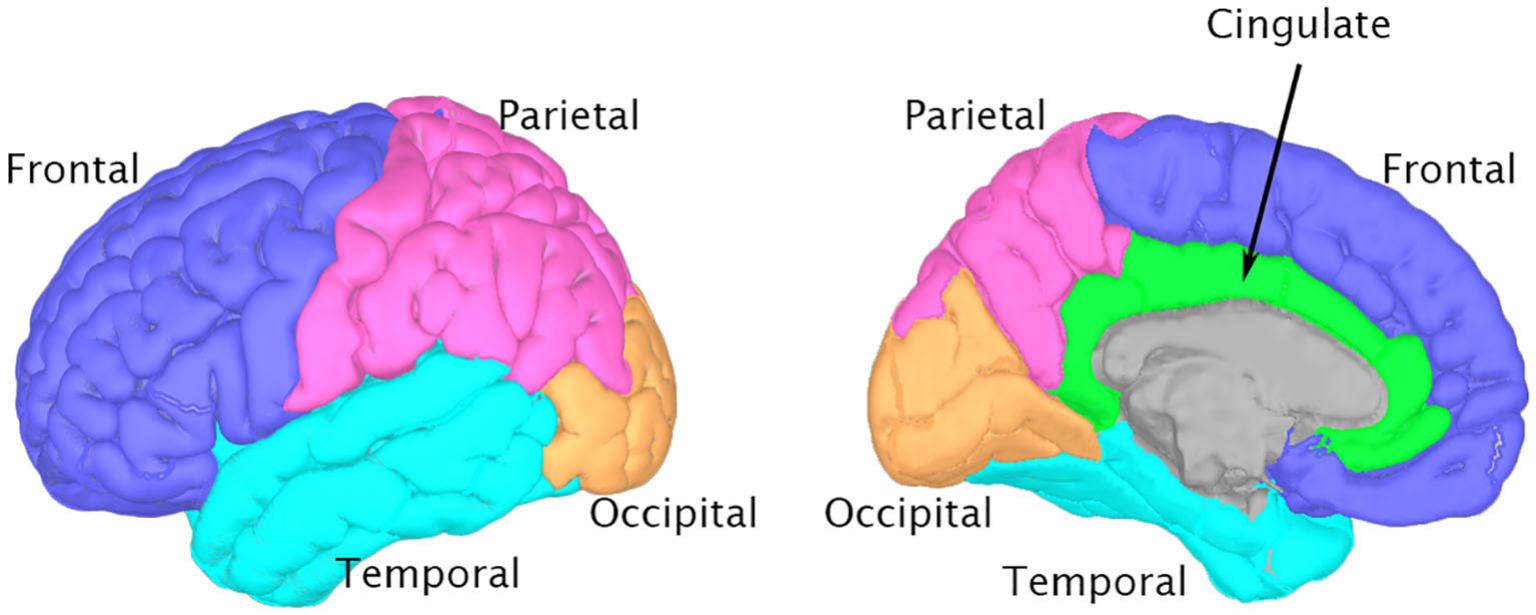
Brain regions for peak alpha frequency Analysis This figure depicts the major brain regions analyzed for peak alpha frequency, based on the Desikan-Killiany atlas. 3D brain models were generated using Brainstorm software (73) and the ICBM152 MRI atlas (74, 75). Left panel: Lateral view highlighting Frontal (blue), Parietal (pink), Temporal (cyan), and Occipital (orange) lobes. Right panel: Medial view showing the Cingulate cortex (green) alongside other visible regions.

The isolated alpha-band spectra were fitted to a Gaussian curve using the least-squares method (59). (**Figure 1E**). The peak of this Gaussian curve was labeled as the PAF (**Figure 1F**). The peak of the curve falling outside the predefined alpha band indicated the absence of a distinct PAF for that region. Such data points were excluded from further analysis. Our method offers definitive identification of PAF, which is beneficial for participants with ambiguous single or double peaks in their data. The PAF was calculated for each region in the Desikan-Killiany atlas. Subsequently, the 68 regions from the atlas were grouped into 10 broader categories corresponding to the cingulate, frontal, occipital, parietal, and temporal regions in both the left and right hemispheres (55) (**Figure 2**). Finally, the PAF values for each of the ten regions were averaged.

### 2.10 Statistical analysis

Differences in age, K-ABC, and SRS scores between the ASD and TD groups were evaluated using two-tailed Student’s t-tests. Chi-square tests were used to assess the differences in sex distribution and the ratio of valid PAFs to the total computed PAFs across the 68 brain regions between the ASD and TD groups. To assess whether the likelihood of obtaining a valid PAF differed by brain region, we performed a chi-square test.

To examine variations in PAF across brain regions and between diagnosis groups (TD vs. ASD), we performed a linear mixed model with PAF as the dependent variable. The fixed effects included region (cingulate, frontal, occipital, parietal, and temporal regions in both hemispheres) and diagnosis (ASD vs. TD), along with their interaction, with subject treated as a random effect.

To investigate the relationship between age and PAF, we conducted separate linear regression analyses for each of the ten predefined regions to predict regional PAF values, with age, ASD diagnosis (TD vs. ASD), and diagnosis-age interaction as predictors. The inclusion of this interaction term was informed by the results of previous studies that indicated possible differential age-associated effects on PAF depending on ASD status (25, 33, 36, 38). The Benjamini-Hochberg procedure (60) was applied for the correction of multiple comparisons controlling the false discovery rate at 0.05. If a significant interaction between age and ASD diagnosis was observed, separate regression analyses were conducted for each group (ASD and TD) to further explore these effects.

To examine the relationship between autistic traits and PAF, separate linear regression analyses were performed for each region, to predict regional PAF from SRS t-scores, ASD diagnosis, and an interaction between SRS t-score and diagnosis. The Benjamini-Hochberg procedure (60) was also applied to correct for multiple comparisons, controlling the false discovery rate at 0.05.

For completeness, we also calculated the relative alpha power for the ten brain regions. Specifically, for each of the 68 regions in the Desikan-Killiany atlas, relative alpha power was calculated as the power within the alpha band (7-13 Hz) divided by the total power across all frequency bands. These values were then averaged for each of the ten predefined regions. To examine whether relative alpha power was stronger in some regions than others and whether this differed by group, we used a mixed-effects model with relative alpha power as the dependent variable. The fixed effects included region (cingulate, frontal, occipital, parietal, and temporal regions in both hemispheres), diagnosis (TD vs. ASD), and their interaction. Subject was included as a random effect.

Additionally, to explore the relationship between cognitive abilities, as measured by the Mental Processing Scale (MPS) and Achievement Scale (ACH), and PAF, we conducted separate linear regression analyses for each region. These analyses predicted regional PAF from MPS or ACH scores, ASD diagnosis, and the interaction between MPS or ACH scores and diagnosis. The Benjamini-Hochberg procedure (60) was also applied to correct for multiple comparisons, controlling the false discovery rate at 0.05.

Prior to any regression analysis, we ascertained that our data adhered to the requisite assumptions. We employed standard procedures to check for linearity, normality, homogeneity of variance, model specifications, and influential points. Notably, some regression models did not satisfy the assumption of homogeneity, necessitating the application of heteroscedasticity-robust standard errors (61). All the statistical analyses were performed using Stata (version 17.0, Stata Corp., College Station, TX, US).

## 3 Results

### 3.1 Participants

Overall, three children were excluded from the analyses: one child diagnosed with ASD showed pronounced psychomotor agitation and was unable to complete the K-ABC assessment; another child with ASD had intellectual disability, reflected in a K-ABC mental processing scale score < 70; and in the TD group, one child exhibited significant body movement during the MEG session, resulting in elevated noise levels in the results. Considering these exclusions, the final sample consisted of 19 children with ASD and 24 TD children. The age range of the children in the ASD group was 60–89 months, whereas that of the children in the TD group was 60–91 months. There were no significant differences in sex, age, number of available epochs, or K-ABC achievement scale scores between the two groups. However, there were significant differences in total SRS score and K-ABC mental processing scale score between the two groups. These findings are summarized in **Table 1**. The mean ADOS-2 Social Affect score for the ASD group was 6.1 (SD = 3.4), the mean ADOS-2 Restricted and Repetitive Behaviors score was 2.1 (SD = 1.4), the mean ADOS-2 total score was 8.2 (SD = 3.8), and the mean ADOS-2 Comparison score was 4.5 (SD = 2.1).

**Table 1:** Characteristics of the participants.

	ASD mean ± SD (min-max)	TD mean ± SD (min-max)	*χ2* or *t*	*p*
*N*	19	24		
Sex (% Male) ^†^	68.4	58.3	0.462	0.497
Age (months) ^‡^	72.5 ± 7.5 (60-89)	69.6 ± 9.0 (60-91)	-1.127	0.266
Epoch number ^‡^	38.6 ± 8.1 (20-52)	42.3 ± 8.0 (24-52)	-1.095	0.140
Total SRS score ^‡^	67.6 ± 14.3 (41-96)	46.4 ± 6.4 (38-68)	-6.493	< 0.001*
K-ABC scores				
MPS ^‡^	103.1 ± 16.7 (77-137)	115.4 ± 12.4 (90-134)	2.619	0.012*
ACH ^‡^	98.0 ± 17.8 (68-134)	107.4 ± 13.7 (84-133)	1.986	0.054

^†^Chi-square test.

^‡^Student’s t-test.

ASD, autism spectrum disorder; TD, typically developing children; K-ABC, Kaufman Assessment Battery for Children; SRS, Social Responsiveness scale; MPS, Mental Processing scale; ACH, achievement score.

The asterisks indicate significant results.

### 3.2 Regional and group differences in PAF

From a total of 2,924 spectral densities (calculated as 68 × (19 + 24)), 2,826 (96.6%) were classified as valid PAFs. Specifically, 1,227 of 1,292 (95.0%) spectral densities were identified as valid PAFs in the ASD group, whereas 1,599 of 1,632 (98.0%) were considered valid PAFs in the TD group. A chi-squared test demonstrated a significant difference between these proportions (χ² = 19.2, p < 0.001), indicating disparities in PAF detection between the groups.

The significant differences in the proportion of valid PAFs across brain regions, as indicated by the chi-square test (χ²(9) = 31.4, p < 0.001), suggest that the likelihood of obtaining a valid PAF varies by brain region. Notably, the frontal and temporal regions had a lower rate of valid PAF estimations compared to the other regions (cingulate, occipital, and parietal regions; **Supplementary Table 1**). This finding aligns with the known distribution of alpha oscillations, which are most prominent in the occipital, parietal, and posterior temporal regions (62).

We used a linear mixed model to analyze the variations in PAF across brain regions and between diagnosis groups (TD vs. ASD). Significant effects were observed in the left occipital region (z = -2.98, p = 0.0028), left temporal region (z = -2.93, p = 0.0034), right occipital region (z = -3.64, p = 0.0003), and right temporal region (z = -2.10, p = 0.036), indicating lower PAF in these regions compared to the left cingulate region (used as a reference). There were no significant main effects of diagnosis or an interaction between region and diagnosis. These findings suggest that while alpha PAF varied across different brain regions, it was similar across TD and ASD groups in our dataset. **Supplementary Table 2** provides a detailed summary of the mixed-effects model results.

### 3.3 Association between PAF and age

We constructed models for each of the ten brain regions (cingulate, frontal, occipital, parietal, and temporal areas in the left and right hemispheres) to investigate the relationship between age and the PAF in each region. These models predicted regional PAF based on age, ASD diagnosis (TD vs. ASD), and an diagnosis-age interaction. Utilizing the Benjamini-Hochberg procedure to correct for multiple comparisons, we observed a significant interaction effect between age and diagnosis in both the right (t(39) = 2.76, p = 0.009) and left (t(39) = 2.93, p = 0.006) cingulate regions. This indicates that the relationship between age and PAF in these regions varies according to ASD status. No significant effects of any predictors were observed in any of the other regions examined. **Table 2** provides a detailed summary of these model findings, highlighting the unique impact of the diagnosis-age interaction on PAF in the cingulate regions.

**Table 2:** Relationship between age and peak alpha frequency.

vs. peak alpha frequency								
Left parietal	Coeff.	Robust SE	*t*	*p*	95% CI		*F*	*R^2^*
**Diagnosis** **(ASD:1, TD:0)**	-2.736	1.308	-2.09	0.043	-5.383	-0.090	0.024	0.203
**Age (months)**	0.010	0.008	1.25	0.217	-0.006	0.027		
**Diagnosis-age interaction**	0.037	0.017	2.09	0.043	0.001	0.073		
Left occipital	Coeff.	Robust SE	*t*	*p*	95% CI		*F*	*R^2^*
**Diagnosis** **(ASD:1, TD:0)**	-2.802	1.618	-1.73	0.091	-6.077	0.473	0.035	0.234
**Age (months)**	0.013	0.008	1.53	0.134	-0.004	0.029		
**Diagnosis-age interaction**	0.041	0.022	1.85	0.072	-0.004	0.086		
Left frontal	Coeff.	Robust SE	*t*	*p*	95% CI		*F*	*R^2^*
**Diagnosis** **(ASD:1, TD:0)**	1.106	2.061	0.54	0.595	-3.063	5.275	0.677	0.021
**Age (months)**	0.011	0.009	1.16	0.253	-0.008	0.043		
**Diagnosis-age interaction**	-0.163	0.291	-0.56	0.581	-0.075	0.043		
Left temporal	Coeff.	Robust SE	*t*	*p*	95% CI		*F*	*R^2^*
**Diagnosis** **(ASD:1, TD:0)**	-1.811	1.883	-0.96	0.342	-5.621	1.997	0.698	0.060
**Age (months)**	0.002	0.010	0.26	0.794	-0.017	0.022		
**Diagnosis-age interaction**	0.026	0.027	0.99	0.329	-0.027	0.080		
Left cingulate	Coeff.	Robust SE	*t*	*p*	95% CI		*F*	*R^2^*
**Diagnosis** **(ASD:1, TD:0)**	-3.847	1.311	-2.93	0.006	-6.498	-1.196	0.007	0.301
**Age (months)**	0.005	0.009	0.56	0.578	-0.013	0.023		
**Diagnosis-age interaction**	0.053	0.018	2.93	0.006*	0.016	0.089		
Right parietal	Coeff.	Robust SE	*t*	*p*	95% CI		*F*	*R^2^*
**Diagnosis** **(ASD:1, TD:0)**	-3.508	1.440	-2.44	0.020	-6.422	-0.595	0.033	0.247
**Age (months)**	0.007	0.007	1.00	0.323	-0.007	0.020		
**Diagnosis-age interaction**	0.048	0.020	2.43	0.020	0.080	0.089		
Right occipital	Coeff.	Robust SE	*t*	*p*	95% CI		*F*	*R^2^*
**Diagnosis** **(ASD:1, TD:0)**	-2.129	1.636	-1.30	0.201	-5.438	1.181	0.102	0.137
**Age (months)**	0.012	0.008	1.60	0.117	-0.003	0.028		
**Diagnosis-age interaction**	0.030	0.023	1.34	0.188	-0.015	0.076		
Right frontal	Coeff.	Robust SE	*t*	*p*	95% CI		*F*	*R^2^*
**Diagnosis** **(ASD:1, TD:0)**	-2.466	1.783	-1.38	0.175	-6.072	1.141	0.228	0.156
**Age (months)**	0.009	0.010	0.83	0.411	-0.012	0.029		
**Diagnosis-age interaction**	0.032	0.025	1.29	0.21	-0.018	0.081		
Right temporal	Coeff.	Robust SE	*t*	*p*	95% CI		*F*	*R^2^*
**Diagnosis** **(ASD:1, TD:0)**	-1.405	1.625	-0.86	0.393	-4.692	1.882	0.273	0.079
**Age (months)**	0.012	0.009	1.37	0.180	-0.006	0.030		
**Diagnosis-age interaction**	0.019	0.023	0.81	0.422	-0.028	0.065		
Right cingulate	Coeff.	Robust SE	*t*	*p*	95% CI		*F*	*R^2^*
**Diagnosis** **(ASD:1, TD:0)**	-3.884	1.406	-2.76	0.009	-6.727	-1.040	0.013	0.311
**Age (months)**	0.006	0.008	0.68	0.501	-0.011	0.023		
**Diagnosis-age interaction**	0.053	0.019	2.76	0.009*	0.014	0.092		

Coeff, regression coefficient; SE, standard error; t: t-statistic; p: p-value; CI, confidence interval; ASD, autism spectrum disorder; TD, typically developing children.

Values in bold with an asterisk indicate significant results obtained after applying the Benjamini-Hochberg procedure to all regression tests.

**Figure 3:**
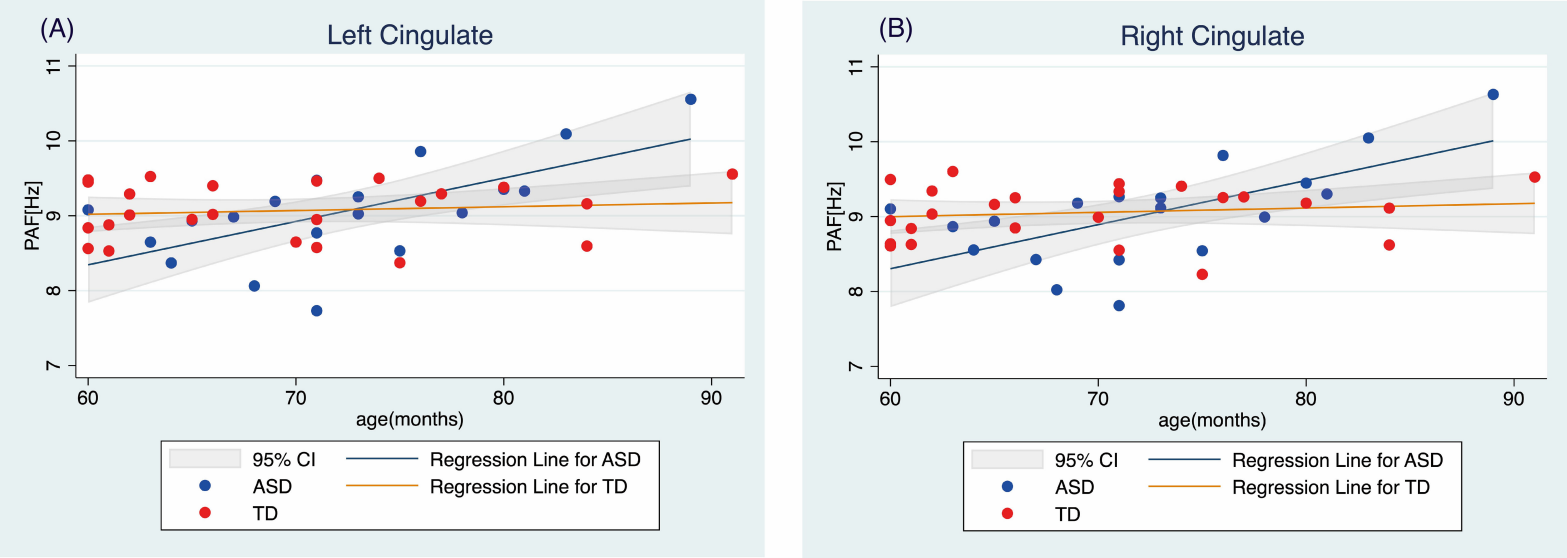
Relationship between age and peak alpha frequency in the **(A)** left and **(B)** right cingulate regions TD, typically developing children; ASD, children with autism spectrum disorder; PAF, peak alpha frequency.

To analyze this interaction effect in the ASD and TD groups further, we performed separate regression analyses to predict the PAF in the right and left cingulate regions based on age. The results indicated that there was no significant correlation between age and PAF in either cingulate region in the TD group. In contrast, a significant relationship between age and PAF was detected in both the left cingulate (t(17)=3.68, p=0.002; **Figure 2**) and right cingulate (t(17)=3.88, p=0.004; **Figure 3**) regions in the ASD group (**Table 3**).

**Table 3:** Regression analysis of the relationship between age and peak alpha frequency.

vs PAF		Coeff.	Robust SE	*t*	*p*	95% CI	*R^2^*
**Left cingulate**	**ASD**	0.58	0.16	3.68	0.002*	0.03 - 0.09	0.42
	**TD**	0.01	0.01	0.56	0.578	-0.01 – 0.02	0.02
**Right cingulate**	**ASD**	0.59	0.02	3.88	0.004*	0.02 – 0.10	0.42
	**TD**	0.01	0.01	0.68	0.502	-0.02 – 0.02	0.02

PAF, peak alpha frequency; Coeff, regression coefficient; SE, standard error; t: t-statistic; p: p-value; CI, confidence interval; ASD, autism spectrum disorder; TD, typically developing children.

Asterisks denote significant results obtained after applying the Benjamini-Hochberg procedure to all regression tests.

### 3.4 Association between PAF and autistic traits

We constructed models for the ten analyzed brain regions to examine the relationship between the PAF in each region and autistic traits measured using the SRS. The models predicted regional PAF based on SRS t-scores, ASD diagnosis (TD vs. ASD), and an interaction between SRS t-scores and diagnosis. Applying the Benjamini-Hochberg procedure for correction of multiple comparisons, we identified a significant effect of SRS t-scores on PAF in the right temporal region (t(39) = 3.17, p = 0.003), indicating that more pronounced autistic traits correspond to higher PAF in this area. No significant effects of any of the predictors were observed in the other regions. **Table 4** provides a detailed summary of these findings, highlighting the specific impact of autistic traits on PAF in the right temporal region of the brain.

**Table 4:** Relationships between PAF and SRS scores in different brain regions.

Vs. Peak alpha frequency								
Left parietal	Coeff.	Robust SE	*t*	*p*	95% CI		*F*	*R^2^*
**Diagnosis** **(ASD:1, TD:0)**	0.65	0.84	0.78	0.440	-1.04	2.35	2.08	0.06
**SRS t-score**	0.02	0.01	2.28	0.028	0.00	0.04		
**Diagnosis-SRS t-score interaction**	-0.02	0.01	-1.22	0.230	-0.05	0.01		
Left occipital	Coeff.	Robust SE	*t*	*p*	95% CI		*F*	*R^2^*
**Diagnosis** **(ASD:1, TD:0)**	-0.08	1.01	-0.08	0.937	-2.12	1.96	1.68	0.10
**SRS t-score**	0.02	0.01	1.80	0.080	0.00	0.03		
**Diagnosis-SRS t-score interaction**	0.00	0.02	-0.02	0.986	-0.03	0.03		
Left frontal	Coeff.	Robust SE	*t*	*p*	95% CI		*F*	*R^2^*
**Diagnosis** **(ASD:1, TD:0)**	-0.22	0.88	-0.25	0.805	-2.00	1.56	0.45	0.03
**SRS t-score**	-0.01	0.01	-0.95	0.035	-0.04	0.01		
**Diagnosis-SRS t-score interaction**	0.01	0.02	0.34	0.732	-0.03	0.04		
Left temporal	Coeff.	Robust SE	*t*	*p*	95% CI		*F*	*R^2^*
**Diagnosis** **(ASD:1, TD:0)**	1.02	1.16	0.88	0.385	-1.33	3.36	0.62	0.03
**SRS t-score**	0.02	0.01	1.28	0.207	-0.01	0.04		
**Diagnosis-SRS t-score interaction**	-0.02	0.02	-0.97	0.336	-0.58	0.02		
Left cingulate	Coeff.	Robust SE	*t*	*p*	95% CI		*F*	*R^2^*
**Diagnosis** **(ASD:1, TD:0)**	-0.29	0.82	-0.36	0.721	-1.95	1.36	0.47	0.03
**SRS t-score**	0.01	0.01	0.69	0.495	-0.01	0.03		
**Diagnosis-SRS t-score interaction**	0.00	0.01	0.15	0.882	-0.03	0.03		
Right parietal	Coeff.	Robust SE	*t*	*p*	95% CI		*F*	*R^2^*
**Diagnosis** **(ASD:1, TD:0)**	0.40	0.76	0.52	0.608	-1.15	1.94	2.07	0.04
**SRS t-score**	0.02	0.01	2.34	0.024	0.00	0.03		
**Diagnosis-SRS t-score interaction**	-0.01	0.01	-0.90	0.373	-0.04	0.01		
Right occipital	Coeff.	Robust SE	*t*	*p*	95% CI		*F*	*R^2^*
**Diagnosis** **(ASD:1, TD:0)**	-0.32	1.11	-0.29	0.774	-2.57	1.93	0.21	0.03
**SRS t-score**	0.00	0.01	0.36	0.721	-0.02	0.03		
**Diagnosis-SRS t-score interaction**	0.00	0.02	0.26	0.793	-0.03	0.04		
Right frontal	Coeff.	Robust SE	*t*	*p*	95% CI		*F*	*R^2^*
**Diagnosis** **(ASD:1, TD:0)**	0.74	0.82	0.90	0.372	-0.913	2.39	0.43	0.03
**SRS t-score**	0.01	0.01	1.39	0.172	-0.01	0.04		
**Diagnosis-SRS t-score interaction**	-0.02	0.01	-1.27	0.213	-0.05	0.01		
Right temporal	Coeff.	Robust SE	*t*	*p*	95% CI		*F*	*R^2^*
**Diagnosis** **(ASD:1, TD:0)**	2.20	1.26	1.76	0.087	-0.33	4.74	3.36	0.13
**SRS t-score**	0.47	0.15	3.17	0.0029*	0.17	0.08		
**Diagnosis-SRS t-score interaction**	-0.48	0.02	-2.22	0.033	-0.91	0.00		
Right cingulate	Coeff.	Robust SE	*t*	*p*	95% CI		*F*	*R^2^*
**Diagnosis** **(ASD:1, TD:0)**	0.23	0.77	0.30	0.763	-1.32	1.78	2.37	0.05
**SRS t-score**	0.02	0.01	2.40	0.021	0.00	0.03		
**Diagnosis-SRS t-score interaction**	-0.01	0.01	-0.72	0.474	-0.03	0.02		

PAF, peak alpha frequency; Coeff, regression coefficient; SE, standard error; t: t-statistic; p: p-value; CI, confidence interval; ASD, autism spectrum disorder; TD, typically developing children; SRS, Social Responsiveness Scale.

Asterisks denote significant results obtained after applying the Benjamini-Hochberg procedure to all regression tests.

**Figure 4:**
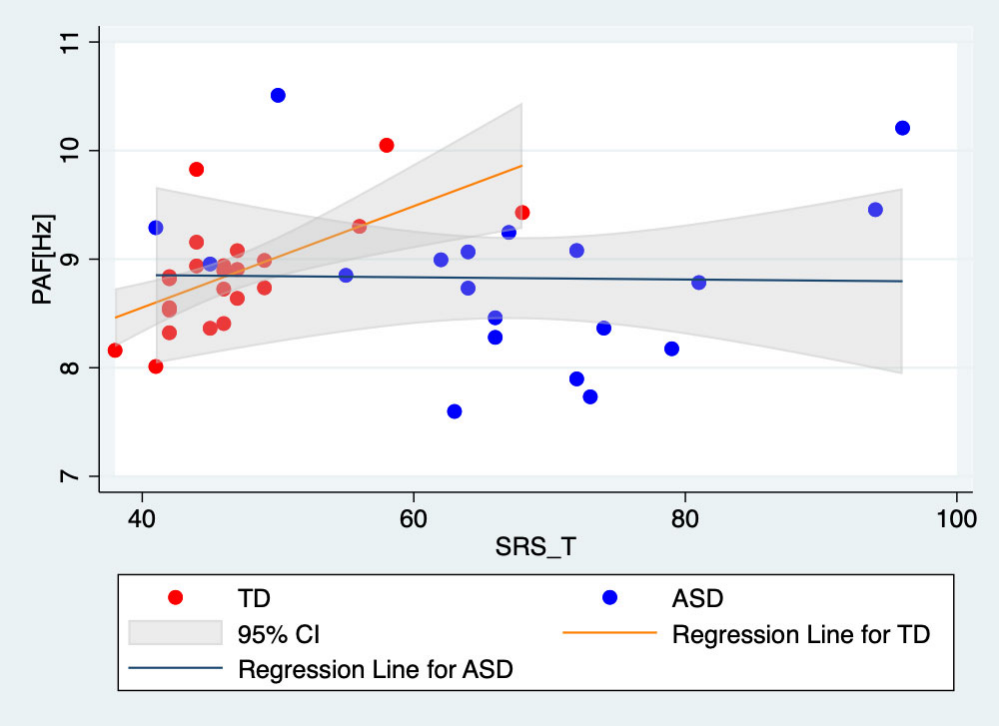
Scatter plots and regression lines for PAF in the right temporal region relative to the SRS T-scores of the TD children and the children with ASD. Red markers and the red regression line represent TD children, highlighting the significant positive correlation between PAF and SRS t-scores in this group. Blue markers and the blue regression line represent the children with ASD, demonstrating the absence of a significant relationship between these variables in the ASD group. Each point represents an individual participant’s data, with the linear fit lines summarizing the trend within each group. This visual comparison underscores the differential impact of autistic traits on neural activity patterns in TD children compared to those with ASD. TD, typically developing children; ASD, children with autism spectrum disorder; PAF, peak alpha frequency.

To further investigate the nature of the significant effect of SRS t-scores on PAF in the right temporal region, we conducted separate linear regression analyses for each group. This approach allowed us to interpret the relationship within each group independently. The results revealed a significant effect of SRS t-scores on PAF in the right temporal region only in the TD group (t(22) = 3.19, p = 0.004); the results for the ASD group was non-significant (t(17) = -0.06, p = 0.950; **Figure 4**). Despite these differences in associations, the interaction term in the combined model was not significant, indicating that the difference in associations between groups did not reach statistical significance.

As an exploratory analysis, we examined the relationship between different types of autistic traits and PAF in TD children. We performed separate linear regression analyses to predict PAF in the right temporal region based on each SRS subscale (i.e., Social Awareness, Social Cognition, Social Communication, Social Motivation, and Autistic Mannerism). Considering the exploratory nature of this analysis, we did not correct for multiple comparisons and set the statistical significance at p < 0.05. The results revealed significant effects for Social Awareness (t(22) = 3.40, p = 0.0026), Social Cognition (t(22) = 2.54, p = 0.019), Social Communication (t(22) = 4.12, p < 0.001), and Autistic Mannerism (t(22) = 3.31, p = 0.003). **Supplementary Table 3** provides a detailed summary of these findings, highlighting the relationships between different aspects of autistic symptoms and PAF in the right temporal region.

### 3.5 Regional and group differences in relative alpha power

To examine whether alpha power was stronger in some regions than others and whether this differed by group, we used a mixed-effects model with alpha power as the dependent variable. The fixed effects included region (cingulate, frontal, occipital, parietal, and temporal regions in both hemispheres), diagnosis (TD vs. ASD), and their interaction. Subject was included as a random effect. We found significant effects of left temporal region (z = -4.52, p < 0.001) and the right cingulate region (z = -2.21, p = 0.0268), indicating that those two regions show lower alpha power compared to left cingulate region (used as a reference). There were no significant main effects of diagnosis or an interaction between region and diagnosis. These findings suggest that while alpha power distribution varied across different brain regions, it was similar across TD and ASD groups in our dataset. **Supplementary Table 4** provides a detailed summary of the mixed-effects model results.

It is noteworthy that the standard errors for the region factor were identical due to the balanced nature of our data, where each participant had measurements for all 10 brain regions.

### 3.6 Association between PAF and intelligence

We constructed models for the ten analyzed brain regions to examine the relationship between PAF in each region and intelligence, as measured by the MPS and ACH scales in the K-ABC. The models predicted regional PAF based on MPS (or ACH) scores, ASD diagnosis (TD vs. ASD), and the interaction between MPS (or ACH) scores and diagnosis. After applying the Benjamini-Hochberg procedure for correction of multiple comparisons, none of the main or interaction effects were significant in any region. These non-significant results suggest that we cannot conclude that intelligence, as measured by the K-ABC, has a strong impact on PAF in either the ASD or TD groups. A detailed summary of the model results is provided in **Supplementary Table 5**.

## 4 Discussion

In this study, we investigated the relationship between PAF and autistic symptoms in young children with ASD and their TD peers. The children, aged 5 to 7 years, underwent MEG in a resting-state, focusing on a fixation cross mark projected onto a screen with their eyes remaining open throughout the recording session. The ASD group was not on any medications and had no other neuropsychiatric disorders. Our analyses revealed no statistically significant differences in PAF between children with ASD and their TD peers across any of the ten examined brain regions. This suggests that, in terms of PAF, children with ASD do not exhibit significant neurophysiological differences in the analyzed regions compared to TD children. This finding is particularly notable as it does not align with our expectation of replicating previous studies that indicated different PAFs in children with ASD within this age range (26, 33). Furthermore, although we hypothesized a significant association between more severe autistic symptoms and higher PAF in both groups, our analysis revealed that more pronounced autistic traits were associated with higher PAF, specifically in the right temporal region, and this relationship was significant only in TD children. This finding differed from our expectations, highlighting the complexity of the relationship between autistic traits and PAF. It is important to note that despite these differences in associations, the interaction term in the combined model was not significant, indicating that the difference in associations between autistic symptoms and PAF across groups did not reach statistical significance.

Interestingly, our results also revealed a nuanced relationship between age and PAF in the cingulate regions of the brain, which varied according to ASD status. Contrary to our hypothesis of a significant association between older age and higher PAF within the TD population, we observed no significant association between age and PAF in the TD group. This finding contrasts with the well-established positive correlation between PAF and age in TD children. This correlation has been consistently reported across various developmental stages, from as early as 12 months, irrespective of familial ASD risk (37), through childhood (5-10 years) (16, 25, 26, 36), and into adolescence (approximately 16 years) (35). Notably, there is a reversal in this trend in adulthood (at approximately 30 years) (34). While recording conditions (i.e., eyes open in a dark room focusing on a fixation cross) could influence the results, the absence of a significant correlation in the present study can likely be attributed to the narrow age range of the TD group (5-7 years), which may not provide sufficient variability to detect an age-PAF association. Additionally, the relatively small sample size and the inherent variability in PAF even among children of the same age likely contributed to this finding. Notably, regions including the left and right occipital showed a tendency towards a positive association between age and PAF (e.g., t = 1.53 for the main effect of age in the left occipital and t = 1.60 for the main effect of age in the right occipital), suggesting a potential underlying trend that warrants further investigation. These region-specific findings highlight the importance of considering anatomical and functional distinctions within the brain when studying neurophysiological markers such as PAF. The differential associations observed across various brain regions may reflect unique aspects of neural maturation and connectivity patterns that are influenced differentially by age and ASD status. Further investigation with a broader age range and larger sample size is necessary to elucidate these complex relationships and their implications for understanding the neurodevelopmental processes underlying ASD.

In the ASD group, we observed a significant association between age and PAF in both the left and right cingulate regions, suggesting an unexpected distinct age-related trajectory of PAF in children with ASD. This relationship is distinct from those reported in the existing literature, which indicate varied associations between age and PAF among individuals with ASD across different age groups. For instance, some studies revealed an association between older age and higher PAF in adolescents with ASD (16.6 ± 5.7 years old) (35) and a converse trend of older age correlating with lower PAF in adults with ASD (30.4 ± 13.6 years old) (34). However, significant associations between age and PAF in younger children, from as early as 12 months to childhood, have not been reported previously (16, 25, 26, 33, 36, 39). It is important to consider the medication status of the participants in these studies because most individuals with ASD are prescribed medications (16, 25, 26, 33–36, 39), adding complexity when comparing our results with those of previous studies (63, 64). Our study is the first to indicate a significant association between older age and higher PAF specifically within the cingulate regions in children aged 5–7 years. To the best of our knowledge, previous studies were not specifically focused on PAF in the cingulate regions. However, research on the power of alpha activity in this region has suggested associations between alpha activity and various factors such as symptoms of pain (65, 66), sleep deprivation (67), and the moderating effects of physical exercise on negative emotions (68). Given that hypersensitivity to pain, sleep disturbances, and emotional dysregulation are prominent characteristics of ASD (69), the discovery of a region-specific association between age and PAF in the cingulate region in children with ASD is particularly compelling. These findings may suggest the potential importance of the cingulate regions as a neurophysiological mechanism underlying some of the core features of ASD. However, further research is needed to explore these associations in larger and more diverse samples. Additional studies are warranted to investigate how these relationships might influence or reflect the developmental trajectory of individuals with ASD.

We observed a significant association between higher PAF in the right temporal region and more severe autistic symptoms, indicated by higher SRS t-scores, exclusively in TD children. This significant association was driven by four of the five SRS subscales: Social Awareness, Social Cognition, Social Communication, and Autistic Mannerism, but not Social Motivation. This finding suggests intriguing possibilities regarding the link between autistic traits and the maturation of the right temporal region in relation to alpha oscillatory development (27, 28, 31, 32). However, it is essential to recognize that the SRS may not capture identical underlying neurophysiological dynamics in TD individuals and in individuals with ASD. For TD children, the SRS could reflect variations in cognitive processing associated with autistic traits, whereas for children with ASD, it may denote specific aspects of ASD pathology. Consequently, the lack of a significant association between PAF and SRS scores in children with ASD may indicate the presence of distinct neurophysiological processes possibly influenced by ASD-specific characteristics such as excitatory/inhibitory imbalances (70). These findings highlight the need for further research to define the neurophysiological factors correlated with SRS scores in both TD and ASD populations and to clarify the relationship between neural markers, such as PAF, and autism-related pathology and broader social information processing constructs.

Overall, while we identified some significant relationships, most of our findings suggest no significant associations between PAF and either age or SRS scores across most brain regions. These null findings highlight the complexity of neurophysiological mechanisms underlying ASD and the need for further research to define the neurophysiological factors correlated with SRS scores in both TD and ASD populations. Future studies should also consider the influence of experimental conditions and the importance of larger, more diverse samples to provide a more comprehensive understanding of these relationships.

This study has several limitations that should be considered when interpreting the results. First, the modest sample size may have limited our ability to detect subtle group differences in PAF. Future studies with larger, more diverse samples are needed to enhance the generalizability of our findings (71). Second, although we excluded children with ASD who were taking medication or had concurrent neuropsychiatric disorders, we did not obtain comprehensive data on other potential comorbidities such as anxiety or ADHD, which could potentially influence EEG measures. Third, our focus on a narrow age range (5-7 years) limits the generalizability of our findings to other developmental stages. Longitudinal studies are needed to understand the trajectory of PAF and its associations with age and autistic traits across the lifespan. Fourth, we relied on a single measure, the SRS score, for the assessment of autistic traits. A multidimensional approach should be employed in future studies to capture the diverse symptomatology of ASD. Fifth, we used a small fixation cross during MEG recordings to help children remain still. Optimally, recordings would have been conducted with eyes closed or in a completely dark room devoid of visual stimulation, similar to the conditions used in the study by Edgar et al. (40). Nevertheless, our approach was necessary to maintain the children’s heads stationary, albeit introducing visual input that could have influenced the results. Sixth, family history of ASD was not screened for in the control group. This could potentially influence the results, as genetic factors associated with ASD might be present in the control group. Seventh, several participants with ASD scored below the clinical cutoff on the SRS, raising concerns about the sensitivity and specificity of the SRS in capturing autistic symptoms in this context. This highlights the inherent challenges in accurately assessing autistic symptoms. While the SRS can be completed by a parent, teacher, or other adult informant, thereby reflecting behaviors observed consistently over weeks or months in natural social contexts, it may not always align perfectly with clinical diagnoses. The SRS assesses social abilities in a variety of real-world communication settings, contrasting with the ADOS, which evaluates behavior in a one-to-one clinical setting. The DISCO, on the other hand, gathers historical information from informants who have known the child since birth. These differences suggest that each assessment tool may capture slightly different aspects of a child’s behavior, contributing to variability in their sensitivity and specificity. Future studies should consider these differences and potentially use multiple assessment tools to provide a more comprehensive evaluation of autistic traits. Finally, although our findings highlight significant age-related differences in PAF in the cingulate regions of children with ASD, the functional implications of these results remain to be fully elucidated. Future research on the link between these neurophysiological markers and specific cognitive, behavioral, and clinical outcomes of ASD are warranted.

In conclusion, this study provides novel insights into the relationship between PAF, age, and autistic traits in young children with ASD and their TD peers. Contrary to our hypotheses, we found no significant differences in PAF across different brain regions between the ASD and TD groups. However, we observed a unique association between age and PAF in the cingulate regions of children with ASD, suggesting a distinct developmental trajectory of alpha oscillations in this population. Additionally, we identified a significant relationship between autistic traits and PAF in the right temporal region, specifically in TD children, indicating a potential role of alpha oscillations in social information processing. These findings highlight the complexity of neurophysiological mechanisms underlying ASD and underscore the importance of considering regional specificity, developmental factors, and the interplay between brain maturation and autistic traits. The absence of significant differences in most regions and the limited age range of our sample suggest that further research with larger, more diverse samples and broader age ranges is needed. Such studies could provide a more comprehensive understanding of the neurophysiological patterns associated with ASD and their potential as biomarkers.

## Data Availability

The raw data supporting the conclusions of this article will be made available by the authors, without undue reservation.
