# Supplementary material for: Relationships between peak alpha frequency, age, and autistic traits in young children with and without autism spectrum disorder
Source: Front Psychiatry. 2024 Aug 30;15:1419815. doi: 10.3389/fpsyt.2024.1419815 (PMC11392836; doi:10.3389/fpsyt.2024.1419815)
Supplement: Supplementary file 1 [file Table1.docx]

Supplementary Material

# Supplementary Tables

**Supplementary Table 1. Valid and Invalid Peak Alpha Frequencies by Brain Region**

| **Brain Region** | **Valid PAF** | **Invalid PAF** |
| --- | --- | --- |
| **Left Cingulate** | 299 | 2 |
| **Left Frontal** | 371 | 16 |
| **Left Occipital** | 168 | 4 |
| **Left Parietal** | 170 | 2 |
| **Left Temporal** | 411 | 19 |
| **Right Cingulate** | 298 | 3 |
| **Right Frontal** | 370 | 17 |
| **Right Occipital** | 167 | 5 |
| **Right Parietal** | 169 | 3 |
| **Right Temporal** | 403 | 27 |
| **Total†** | 2826 | 98 |

The table shows the distribution of valid and invalid peak alpha frequency (PAF) calculations across different brain regions.

Valid PAF: Number of valid PAF calculations; Invalid PAF: Number of invalid PAF calculations. Brain regions: Left/Right Cingulate, Frontal, Occipital, Parietal, and Temporal.

† Overall chi-square test across all regions (χ2 = 31.3577, p < 0.001)

**Supplementary Table 2. Effects of Diagnosis and Brain Region on Peak Alpha Frequency (PAF): Multivariate Regression Analysis Results**

| **Variable** | **Coeff.** | **Std. Err.** | ***z*** | ***p*** | **95% CI** | |
| --- | --- | --- | --- | --- | --- | --- |
| **Diagnosis** | <0.001 | 0.175 | <0.001 | 0.999 | -0.344 | 0.343 |
| **Region** |  |  |  |  |  |  |
| **Left Cingulate (Reference)** |  |  |  |  |  |  |
| **Left Frontal** | -0.011 | 0.103 | -0.110 | 0.914 | -0.213 | 0.190 |
| **Left Occipital** | -0.307 | 0.103 | -2.980 | 0.003 | -0.508 | -0.105 |
| **Left Parietal** | -0.139 | 0.103 | -1.350 | 0.176 | -0.341 | 0.062 |
| **Left Temporal** | -0.301 | 0.103 | -2.930 | 0.003 | -0.502 | -0.099 |
| **Right Cingulate** | -0.013 | 0.103 | -0.130 | 0.899 | -0.215 | 0.189 |
| **Right Frontal** | -0.018 | 0.103 | -0.170 | 0.862 | -0.219 | 0.184 |
| **Right Occipital** | -0.375 | 0.103 | -3.640 | <0.001 | -0.576 | -0.173 |
| **Right Parietal** | -0.141 | 0.103 | -1.370 | 0.171 | -0.342 | 0.061 |
| **Right Temporal** | -0.216 | 0.103 | -2.100 | 0.036 | -0.417 | -0.014 |
| **Interaction** |  |  |  |  |  |  |
| **Diagnosis * Left Cingulate (Reference)** |  |  |  |  |  |  |
| **Diagnosis * Left Frontal** | -0.101 | 0.155 | -0.660 | 0.512 | -0.405 | 0.202 |
| **Diagnosis * Left Occipital** | 0.152 | 0.156 | 0.980 | 0.329 | -0.153 | 0.458 |
| **Diagnosis * Left Parietal** | -0.015 | 0.155 | -0.090 | 0.925 | -0.318 | 0.289 |
| **Diagnosis * Left Temporal** | 0.101 | 0.155 | 0.650 | 0.513 | -0.202 | 0.405 |
| **Diagnosis * Right Cingulate** | -0.015 | 0.155 | -0.100 | 0.922 | -0.318 | 0.288 |
| **Diagnosis * Right Frontal** | -0.197 | 0.155 | -1.280 | 0.202 | -0.501 | 0.106 |
| **Diagnosis * Right Occipital** | 0.100 | 0.155 | 0.650 | 0.518 | -0.203 | 0.403 |
| **Diagnosis * Right Parietal** | 0.016 | 0.155 | 0.110 | 0.915 | -0.287 | 0.320 |
| **Diagnosis * Right Temporal** | -0.026 | 0.155 | -0.170 | 0.866 | -0.329 | 0.277 |

Coeff.: Coefficient; Std. Err.: Standard Error; *z*: z-score; *p*: p-value; 95% CI: 95% Confidence Interval; Diagnosis: Typically Developing Children (reference) vs. Autism Spectrum Disorder; Region: Brain regions (Left Cingulate as the reference); Interaction: Diagnosis * Region

**Supplementary Table 3. Relationship Between SRS Subscales and Peak Alpha Frequency in Typically Developing Children and Children with Autism Spectrum Disorder**

|  |  | **Coeff.** | **Robust SE** | ***t*** | ***p*** | **95% CI** | | ***R^2^*** |
| --- | --- | --- | --- | --- | --- | --- | --- | --- |
|  | SRS subscale |  |  |  |  |  |  |  |
| TD | Social Awareness | 0.03 | 0.01 | 3.40 | 0.003 | 0.01 | 0.05 | 0.36 |
|  | Social Cognition | 0.02 | 0.01 | 2.54 | 0.019 | 0.00 | 0.04 | 0.11 |
|  | Social Communication | 0.04 | 0.01 | 4.12 | <0.001 | 0.02 | 0.07 | 0.41 |
|  | Social Motivation | 0.01 | 0.02 | 0.35 | 0.727 | -0.03 | 0.04 | 0.01 |
|  | Autistic Mannerism | 0.04 | 0.01 | 3.31 | 0.003 | 0.02 | 0.07 | 0.36 |
| ASD | Social Awareness | -0.03 | 0.02 | -1.48 | 0.156 | -0.06 | 0.01 | 0.13 |
|  | Social Cognition | -0.01 | 0.01 | -0.74 | 0.471 | -0.04 | 0.02 | 0.03 |
|  | Social Communication | 0.00 | 0.02 | 0.15 | 0.883 | -0.03 | 0.04 | 0.00 |
|  | Social Motivation | 0.01 | 0.01 | 0.47 | 0.646 | -0.02 | 0.04 | 0.02 |
|  | Autistic Mannerism | 0.00 | 0.02 | 0.27 | 0.792 | -0.03 | 0.04 | 0.01 |

Coeff.: Coefficient; Robust SE: Robust Standard Error; *t*: t-statistic; *p*: p-value; 95% CI: 95% Confidence Interval; *R^2^*^:^ R-squared value; SRS: Social Responsiveness Scale; TD: Typically Developing Children; ASD: Autism Spectrum Disorder

**Supplementary Table 4: Mixed-effects regression analysis of alpha power across brain regions and diagnostic groups**

| **Variable** | **Coeff.** | **Std. Err.** | ***z*** | ***p*** | **95% CI** | |
| --- | --- | --- | --- | --- | --- | --- |
| **Diagnosis** | -0.006 | 0.007 | -0.780 | 0.436 | -0.020 | 0.009 |
| **Region** |  |  |  |  |  |  |
| **Left Cingulate (Reference)** |  |  |  |  |  |  |
| **Left Frontal** | <0.001 | 0.003 | -0.130 | 0.893 | -0.007 | 0.006 |
| **Left Occipital** | -0.001 | 0.003 | -0.220 | 0.827 | -0.007 | 0.006 |
| **Left Parietal** | -0.002 | 0.003 | -0.490 | 0.628 | -0.008 | 0.005 |
| **Left Temporal** | -0.015 | 0.003 | -4.520 | <0.001 | -0.022 | -0.009 |
| **Right Cingulate** | -0.008 | 0.003 | -2.210 | 0.027 | -0.014 | -0.001 |
| **Right Frontal** | -0.003 | 0.003 | -0.790 | 0.427 | -0.009 | 0.004 |
| **Right Occipital** | 0.002 | 0.003 | 0.630 | 0.526 | -0.005 | 0.009 |
| **Right Parietal** | 0.002 | 0.003 | 0.550 | 0.584 | -0.005 | 0.009 |
| **Right Temporal** | 0.006 | 0.003 | 1.760 | 0.078 | -0.001 | 0.013 |
| **Interaction** |  |  |  |  |  |  |
| **Diagnosis * Left Cingulate (Reference)** |  |  |  |  |  |  |
| **Diagnosis * Left Frontal** | -0.002 | 0.005 | -0.400 | 0.686 | -0.012 | 0.008 |
| **Diagnosis * Left Occipital** | -0.002 | 0.005 | -0.310 | 0.755 | -0.012 | 0.008 |
| **Diagnosis * Left Parietal** | -0.005 | 0.005 | -1.050 | 0.294 | -0.015 | 0.005 |
| **Diagnosis * Left Temporal** | <0.001 | 0.005 | 0.080 | 0.935 | -0.010 | 0.010 |
| **Diagnosis * Right Cingulate** | <0.001 | 0.005 | -0.090 | 0.924 | -0.011 | 0.010 |
| **Diagnosis * Right Frontal** | -0.006 | 0.005 | -1.100 | 0.273 | -0.016 | 0.004 |
| **Diagnosis * Right Occipital** | -0.002 | 0.005 | -0.460 | 0.644 | -0.012 | 0.008 |
| **Diagnosis * Right Parietal** | 0.002 | 0.005 | 0.380 | 0.706 | -0.008 | 0.012 |
| **Diagnosis * Right Temporal** | -0.002 | 0.005 | -0.410 | 0.682 | -0.012 | 0.008 |

It is noteworthy that the standard errors for the region factor were identical due to the balanced nature of our data, where each participant had measurements for all 10 brain regions. Coeff.: Coefficient; Std. Err.: Standard Error; *z*: z-score; *p*: p-value; 95% CI: 95% Confidence Interval; Region: Brain regions (Left Cingulate as reference); Interaction: Diagnosis * Region.

**Supplementary Table 5: Regression analysis of alpha peak frequency across brain regions, diagnostic groups, and K-ABC scores**

| **Variable** | **Coeff.** | **Std. Err.** | ***t*** | ***p*** | **95% CI** | |
| --- | --- | --- | --- | --- | --- | --- |
| **Diagnosis** |  |  |  |  |  |  |
| **Left Cingulate** | -1.155 | 1.034 | -1.12 | 0.271 | -3.245 | 0.936 |
| **Left Frontal** | -1.801 | 1.263 | -1.43 | 0.162 | -4.356 | 0.754 |
| **Left Occipital** | -1.585 | 1.276 | -1.24 | 0.222 | -4.168 | 0.997 |
| **Left Parietal** | -1.135 | 1.092 | -1.04 | 0.305 | -3.343 | 1.073 |
| **Left Temporal** | -2.263 | 1.099 | -2.06 | 0.046 | -4.485 | -0.040 |
| **Right Cingulate** | -1.171 | 0.971 | -1.21 | 0.235 | -3.135 | 0.794 |
| **Right Frontal** | -1.028 | 1.019 | -1.01 | 0.319 | -3.089 | 1.033 |
| **Right Occipital** | -1.823 | 1.114 | -1.64 | 0.110 | -4.076 | 0.430 |
| **Right Parietal** | -1.513 | 1.054 | -1.44 | 0.159 | -3.646 | 0.620 |
| **Right Temporal** | -1.688 | 0.945 | -1.79 | 0.082 | -3.599 | 0.224 |
| **KABC_ACH** |  |  |  |  |  |  |
| **Left Cingulate** | 0.001 | 0.006 | 0.12 | 0.907 | -0.011 | 0.012 |
| **Left Frontal** | -0.001 | 0.009 | -0.10 | 0.924 | -0.019 | 0.017 |
| **Left Occipital** | -0.002 | 0.007 | -0.24 | 0.815 | -0.016 | 0.013 |
| **Left Parietal** | 0.005 | 0.006 | 0.82 | 0.418 | -0.007 | 0.017 |
| **Left Temporal** | -0.003 | 0.006 | -0.43 | 0.669 | -0.014 | 0.009 |
| **Right Cingulate** | 0.002 | 0.005 | 0.37 | 0.714 | -0.008 | 0.012 |
| **Right Frontal** | 0.010 | 0.005 | 1.79 | 0.081 | -0.001 | 0.020 |
| **Right Occipital** | 0.004 | 0.006 | 0.63 | 0.535 | -0.009 | 0.017 |
| **Right Parietal** | 0.001 | 0.006 | 0.18 | 0.860 | -0.011 | 0.013 |
| **Right Temporal** | 0.003 | 0.006 | 0.60 | 0.553 | -0.008 | 0.015 |
| **KABC_MEN** |  |  |  |  |  |  |
| **Left Cingulate** | 0.002 | 0.006 | 0.36 | 0.718 | -0.010 | 0.015 |
| **Left Frontal** | -0.004 | 0.009 | -0.41 | 0.685 | -0.023 | 0.015 |
| **Left Occipital** | -0.009 | 0.009 | -0.96 | 0.345 | -0.027 | 0.010 |
| **Left Parietal** | 0.003 | 0.009 | 0.38 | 0.708 | -0.014 | 0.021 |
| **Left Temporal** | -0.007 | 0.009 | -0.77 | 0.444 | -0.024 | 0.011 |
| **Right Cingulate** | 0.003 | 0.006 | 0.49 | 0.624 | -0.009 | 0.014 |
| **Right Frontal** | 0.008 | 0.006 | 1.27 | 0.211 | -0.005 | 0.021 |
| **Right Occipital** | -0.005 | 0.009 | -0.56 | 0.580 | -0.024 | 0.014 |
| **Right Parietal** | -0.001 | 0.006 | -0.11 | 0.912 | -0.013 | 0.012 |
| **Right Temporal** | 0.004 | 0.011 | 0.34 | 0.737 | -0.019 | 0.026 |
| **Interaction (Diagnosis * KABC_ACH)** |  |  |  |  |  |  |
| **Left Cingulate** | 0.012 | 0.010 | 1.25 | 0.220 | -0.007 | 0.031 |
| **Left Frontal** | 0.017 | 0.012 | 1.47 | 0.149 | -0.006 | 0.041 |
| **Left Occipital** | 0.018 | 0.012 | 1.51 | 0.138 | -0.006 | 0.042 |
| **Left Parietal** | 0.012 | 0.010 | 1.19 | 0.240 | -0.008 | 0.032 |
| **Left Temporal** | 0.024 | 0.010 | 2.28 | 0.028 | 0.003 | 0.045 |
| **Right Cingulate** | 0.012 | 0.009 | 1.35 | 0.184 | -0.006 | 0.030 |
| **Right Frontal** | 0.009 | 0.009 | 1.02 | 0.315 | -0.009 | 0.028 |
| **Right Occipital** | 0.020 | 0.011 | 1.89 | 0.066 | -0.001 | 0.041 |
| **Right Parietal** | 0.016 | 0.010 | 1.62 | 0.114 | -0.004 | 0.035 |
| **Right Temporal** | 0.017 | 0.009 | 1.86 | 0.070 | -0.001 | 0.036 |
| **Interaction (Diagnosis * KABC_MEN)** |  |  |  |  |  |  |
| **Left Cingulate** | -0.001 | 0.011 | -0.12 | 0.904 | -0.023 | 0.021 |
| **Left Frontal** | 0.016 | 0.012 | 1.34 | 0.187 | -0.008 | 0.039 |
| **Left Occipital** | 0.008 | 0.013 | 0.59 | 0.560 | -0.019 | 0.035 |
| **Left Parietal** | -0.000 | 0.012 | -0.04 | 0.969 | -0.025 | 0.024 |
| **Left Temporal** | 0.021 | 0.012 | 1.78 | 0.082 | -0.003 | 0.044 |
| **Right Cingulate** | -0.001 | 0.011 | -0.13 | 0.894 | -0.023 | 0.020 |
| **Right Frontal** | -0.001 | 0.012 | -0.08 | 0.937 | -0.026 | 0.024 |
| **Right Occipital** | 0.011 | 0.013 | 0.91 | 0.369 | -0.014 | 0.037 |
| **Right Parietal** | 0.002 | 0.012 | 0.16 | 0.872 | -0.022 | 0.025 |
| **Right Temporal** | 0.008 | 0.013 | 0.59 | 0.562 | -0.019 | 0.035 |

Separate regression analyses were conducted for each of the 10 brain regions. Interaction terms represent the differential effect of K-ABC scores on alpha peak frequency between ASD and TD groups.

Coeff.: Coefficient; Robust SE: Robust Standard Error; *t*: t-statistic; *p*: p-value; 95% CI: 95% Confidence Interval; K-ABC: Kaufman Brief Intelligence Test; ACH: Achievement Scale; MEN: Mental Processing Scale; TD: Typically Developing Children; ASD: Autism Spectrum Disorder.
